# The impact of an MSU service on acute stroke care in a middle-sized city: a simulation-based analysis

**DOI:** 10.1007/s00415-024-12515-w

**Published:** 2024-06-20

**Authors:** Kristina Szabo, Till Nagel, Alexander Grund, Alexander Kravatzky, Vesile Sandikci, Markus Radder, Johann Rink, Carolin Hoyer

**Affiliations:** 1 Department of Neurology, Medical Faculty Mannheim, Heidelberg University, Theodor-Kutzer-Ufer 1-3, 68167 Mannheim, Germany; 2 Human Data Interaction Lab, Mannheim University of Applied Sciences, Mannheim, Germany; 3 Department of Radiology and Nuclear Medicine, Mannheim University Medical Centre, Heidelberg University, Mannheim, Germany

Sirs,

Intravenous thrombolysis with alteplase is an effective but highly time-sensitive therapy for acute ischemic stroke; thrombolysis within one hour of onset (the so-called "golden hour") is associated with the best clinical outcomes, but only 1.4% of patients are treated this early [6, 13]. Although in-hospital acute stroke care in urban areas in Germany has continued to improve in recent years, particularly with an increase in the number and access to mechanical thrombectomy, rates of intravenous thrombolysis have remained stable [12]. This ceiling effect may indicate the need for further focus on optimizing prehospital management of acute stroke patients. Perhaps the most exciting approach to reducing prehospital delay is the use of specialized ambulances equipped with stroke care teams, CT scanners and point-of-care laboratory testing devices—Mobile Stroke Units (MSUs)—to provide diagnosis and treatment of acute stroke in the field. Current guidelines of the European Stroke Organization recommend the use of MSUs over conventional care for the prehospital management of patients with suspected stroke because of their positive impact on various dimensions of stroke care, such as IVT rates and short- and long-term stroke outcomes [14]. Due to associated high dispatch volumes, large and densely populated cities have been considered ideal for MSU utilization. Moreover, MSUs may enhance care in remote rural areas often lacking medical services [5]. However, the identification of the most suitable environment for specific ways of MSU implementation—urban or rural—as well as the transferability of the concept to new settings still remains largely unanswered questions. A recent study investigated the potential effect of an MSU in the German–Danish border territory, where different healthcare—systemic and organizational principles—apply [1].

Prior to launching an MSU service in the city of Mannheim, a middle-sized urban region with 310,000 inhabitants and a surface area of 145 square kilometers, we sought to estimate potential benefits of MSU utilization for acute stroke care in such a setting in Germany, which has not yet been put under scientific scrutiny.

In this retrospective cohort study, we analyzed data of 552 patients with complete data regarding prehospital process times and a confirmed diagnosis of acute cerebrovascular events (CVE), including ischemic and hemorrhagic strokes and transient ischemic attacks, admitted to the Comprehensive Stroke Center, Department Neurology, University Hospital Mannheim, Germany between 06/2022 and 06/2023. From a total of 1,051 cases extracted from our Comprehensive Stroke Center database, the following were excluded: secondary transports to our hospital (*N* = 147), in-house strokes (*N* = 19), self-presenting patients (*N* = 132), unknown or extended time windows (*N* = 14), and incomplete documentation of prehospital process times (*N* = 187). We compared real-world emergency medical care (EMC) data with a modeled scenario in which an MSU is stationed at our hospital, excellently accessible and centrally located within Mannheim’s geographical boundaries.

Process times for patients receiving IVT were determined as follows:

for EMC care:

Alarm-to-patient time = timespan between alarm and arrival on scene

Alarm-to-needle time = timespan between alarm and arrival a hospital + door-to-needle time

Onset-to-needle time = timespan between onset and alarm + alarm-to-needle time

for simulated MSU care:

Alarm-to-patient time = travel time from hospital to scene

Alarm-to-needle time = travel time from hospital to scene + door-to-needle time

Onset-to-needle time = timespan between onset and alarm + alarm-to-needle time

Process times for all other patients were determined as follows:

for EMC care:

Alarm-to-patient time = timespan between alarm and arrival on scene

Alarm-to-stroke-unit-admission time = timespan between alarm and arrival a hospital + mean door-to-needle time (assumed to represent average work-up time of stroke patients in the emergency department)

for simulated MSU care:

Alarm-to-patient time = travel time from hospital to scene

Alarm-to-stroke-unit-admission time = travel time from hospital to scene + mean door-to-needle time + travel time from scene to hospital

For the comparison of the intra-subject difference in process times for EMC care and for simulated MSU care, we used paired sample *t* tests. In addition, the rate of thrombolysis performed within 1 h after onset was calculated and possible differences between the two scenarios concerning the golden-hour thrombolysis rates were calculated using the chi^2^-test. *P* < 0.05 indicates statistical significance. Statistical analysis was performed using IBM SPSS Statistics Version 29.

MSU travel times were determined by identifying the optimal routes via Geoapify APIs. Case locations were placed using a Gaussian jittering method within a 500-m radius to enhance patient privacy [15]. The study was approved by the local ethics committee.

**Fig. 1:**
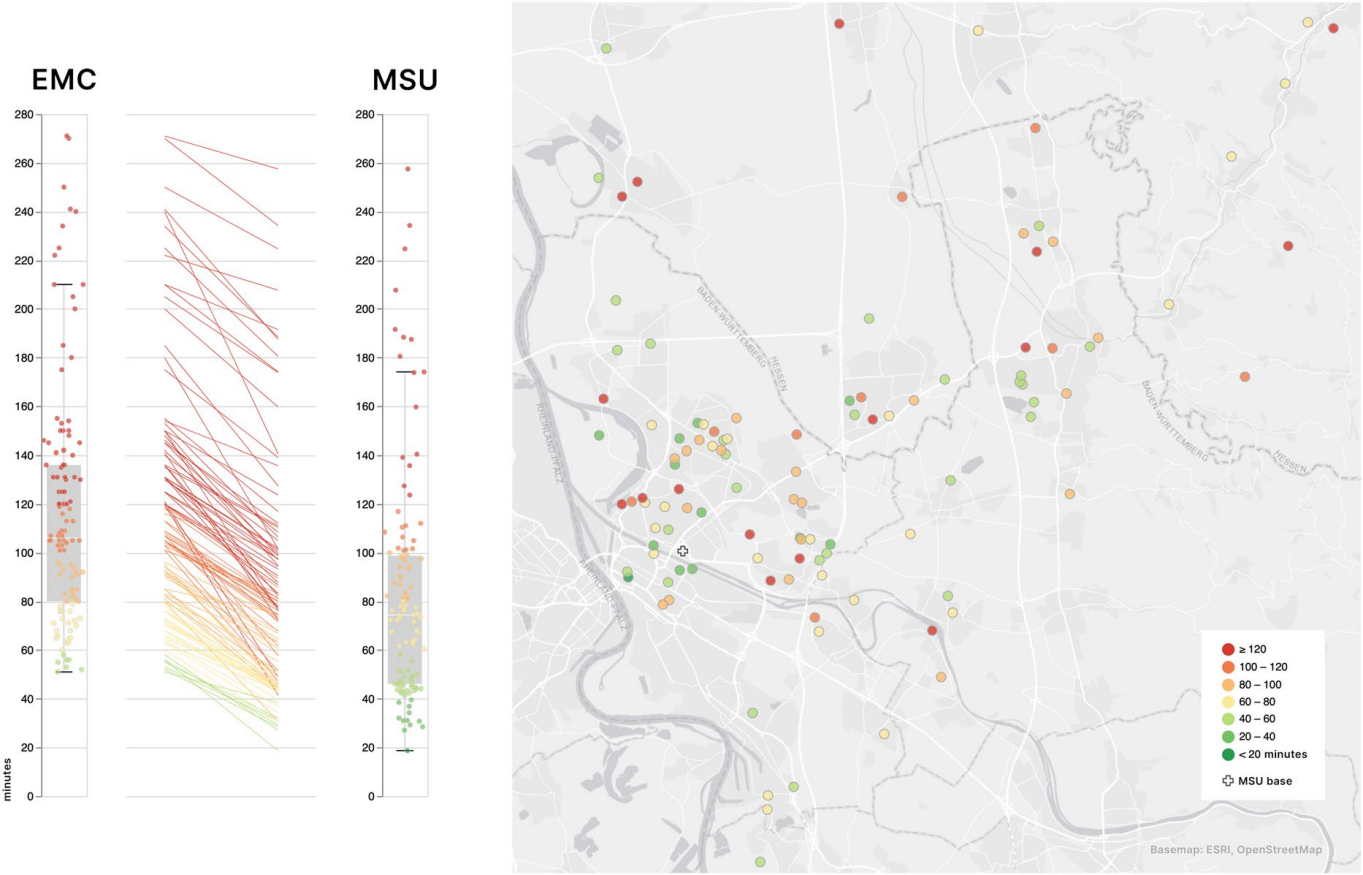
This figure illustrates the spatial and temporal distribution of stroke response cases. On the left, two dot strip plots overlaid on box plots reveal the onset-to-needle times for all cases in both real-world EMC and modeled MSU settings, with a center slope chart depicting the time saved per case, emphasizing the reduced median times in the MSU scenario. The map on the right details case locations in Mannheim and surroundings, marking the MSU base with a cross. Color coding throughout reflects onset-to-needle times: green tones indicate times under the critical 60-min 'golden hour,' while red tones denote longer times

Even though the MSU requires more time to reach the scene—differing by 4 min to EMC—alarm-to-needle time in thrombolysed patients is accelerated by 35 min in this setting (Table 1, Fig. 1). In addition, the rate of patients thrombolysed within 60 min from symptom onset is significantly higher when an MSU is utilized: MSU care increases the proportion of golden-hour thrombolysis more than fivefold (MSU 43/116; 37.1% versus EMC 8/116; 6.9%). All other CVE patients are transferred to dedicated neurological in-hospital care significantly faster in the MSU scenario (Table 1).

**Table 1:** Comparison of real-world EMC care versus MSU service process times

		**EMC**	**MSU**	**p-value**
**Thrombolysed strokes,** M (SD), min	alarm-to-patient	9.98 (4.81)	13.20 (7.06)	** < 0.001**
**[n = 116]**	alarm-to-needle	80.18 (23.98)	45.48 (17.86)	** < 0.001**
**CVEs, no IVT,** M (SD), min	alarm-to-patient	10.10 (6.67)	13.89 (7.03)	** < 0.001**
**[n = 436]**	alarm-to-stroke unit	84.30 (20.81)	59.63 (14.51)	** < 0.001**

CVE: cerebrovascular events; EMC: emergency medical service; IVT: intravenous thrombolysis; MSU: mobile stroke unit; M: mean; SD: standard deviation

Using historical data from previous years of EMC-supported stroke care in a middle-sized urban region in Germany, we demonstrate a positive impact of simulated MSU-based care. Longer driving times of the MSU to the scene are likely related to its set dispatch location at the hospital, while routinely the EMC vehicle closest to the scene is deployed by the dispatch center. However, the expedition of further downstream process components presumably more than counterbalances this initial delay. Even though the optimal setting for MSUs remains uncertain, examples from MSU programs conducted in various urban and rural regions worldwide demonstrate that MSU services can be successfully adapted to align with each region-specific stroke system of care, thereby providing time-efficient expertise in the field. In accordance with this assumption, our data indicate that an MSU service in Mannheim may drastically expedite process times for all CVE patients with a reduction of onset-to-needle and alarm-to-needle times in patients eligible for thrombolysis, as well as faster admission of all patients with completed emergency work-up to a stroke unit. The resulting circumvention of the hospital’s emergency department (ED) brings additional distinct advantages besides shortening care pathway times, such as a reduction in ED-associated delays, adverse events, and patients’ risks particularly in situations of crowding [8, 11] as well as more efficient use of scarce ED resources. Interestingly, MSU accelerates processes in every single case of our cohort regardless of patient location. As a consequence, in our use scenario, the MSU should be dispatched whenever possible and not only to patients beyond a certain radius around the dispatch location. Of note, a more than fivefold increase in golden-hour thrombolysis is expected, which is similar to previously reported data [3]. In Germany, the IVT rate averages at 16.3%. This is approximately twice as high as the European mean [12] and thus indicates an already well-established and well-functioning system for acute stroke care. We are optimistic that the implementation of MSU-based prehospital stroke care will improve IVT rates even further, in particular, we expect a leftward shift of the onset-to-needle time distribution, ideally below 60 min.

Despite the substantial resources required for MSU operation, economic data from various MSU sites indicate that the avoidance of disability has the potential to render the public investment in MSUs cost-effective [7]. Although MSUs have been demonstrated to effectively increase IVT rates, golden-hour thrombolysis rates, and to improve stroke outcomes, their superiority for patients requiring mechanical thrombectomy has not yet been established [9]. Potential explanations for this discrepancy include differences in MSU protocols concerning the capability of and indication for CT angiography, routes of transport to the angiography suite, and other aspects of local stroke systems of care. Future clinical research is needed to address additional measures to optimize the prehospital workflow, which has been a relatively neglected aspect of the stroke response chain. In addition to prehospital treatment with MSUs, these include the development of strategies to improve education of EMS personnel and the general public, the introduction of triage and prenotification tools, and the implementation of novel therapeutic approaches such as neuroprotective agents [2, 4].

Our results should be interpreted in the context of some limitations. First, the reliance on calculated optimal routes for MSU deployment in the simulation rather than actual travel paths, and thus the lack of variables such as traffic congestion or adverse weather conditions that could affect travel times, may affect the validity of the findings. Second, this retrospective cohort study is critically dependent on the accuracy and completeness of patient records and only included patients with complete prehospital documentation. Given the high number of patients with incomplete data, this may have introduced a bias. Finally, the study was conducted as a single-center analysis; therefore, the results may not be generalizable to other settings with different organizational structures.

While this simulation and the subsequent de novo implementation of an MSU service in the city of Mannheim are the first steps in this endeavor, whose financial feasibility has already been demonstrated [10], the meaningful expansion of the catchment area will subsequently follow. Our findings suggest that MSU utilization is generally scalable to and beneficial for novel scenarios beyond those already established, which in turn will deliver important lessons for the continuous refinement of MSU-related organizational and procedural aspects.
